# Supplementary material for: Trajectories toward maximum power and inequality in resource distribution networks
Source: PLoS One. 2020 Mar 10;15(3):e0229956. doi: 10.1371/journal.pone.0229956 (PMC7064246; doi:10.1371/journal.pone.0229956)
Supplement: S1 Text — (DOCX) [file pone.0229956.s001.docx]

**S1 Text. Maximum power derivation**

Recall from Eq. 2, assuming that the network is flow-preserving, such that the sum of all resource flows into the system is equal to the sum of the resource flows across all consumers, the equation for power consumption at the consumer nodes$P_{C}$is:

$P_{C}=I_{C}V_{C}=\left( \frac{V_{R}-V_{C}}{R} \right)V_{C}=\left( \frac{V_{R}V_{C}-V_{C}^{2}}{R} \right) .$ (9)

To find the maximum power, the first derivative of power with respect to potential is taken and set to zero, and the equation is solved to find the critical points:

$\frac{\partial P_{C}}{\partial V_{C}}=\left( -RV_{R} \right)+2RV_{C}=0 ,$ (10a)

$V_{C}=\frac{V_{R}}{2} ,$ (10b)

While this is most simply illustrated in the case of power transfer between two nodes on a single link, complex networks such as the ones in view here can be simplified using an algorithm such as Thévenin’s theorem. Although an explicit Thévenin equivalent was not computed for the networks here, their dynamics could be mapped to one, such that at maximum power,

$\bar{V_{C}}=\frac{\bar{V_{R}}}{2} ,$ (11)

This also implies that *R* in these equations is a mean term for the characteristics of all links across the network, or$\bar{R}$, which is discussed further in the text as$R_{E}$.
