# Supplementary material for: Trajectories toward maximum power and inequality in resource distribution networks
Source: PLoS One. 2020 Mar 10;15(3):e0229956. doi: 10.1371/journal.pone.0229956 (PMC7064246; doi:10.1371/journal.pone.0229956)
Supplement: S1 Table — Parameterisation and power consumption details of networks simulated: a) branched networks, and b) random and radial burst networks. The slope of the linear relationship between σPC and I2 shown in Fig 1 was calculated for each plot using least-squares regression, and compared to the value of σRE calculated using Eq 5 and the consumer and resource potentials for each network, shown in the table here. The least-squares regression estimate of σRE is shown in brackets below the original estimate using consumer and resource potentials, for the networks plotted. (DOCX) [file pone.0229956.s002.docx]

| **Topology** | **Link strength** | **No. of consumers** | **Branch points** | **Avg. consumer potential at maximum power** | **Avg. resource potential at maximum power** | **Maximum power** | **$\sigma_{R_{E}}$** |
| --- | --- | --- | --- | --- | --- | --- | --- |
| Plane | Proportional | 510 | 0 | 1267.963 | 2560 | 3362638.240 | 10.117 |
| Plane | Proportional | 256 | 0 | 1280.465 | 2560 | 1802894.140 | 10.380 |
| Plane | Proportional | 256 | 2 | 1291.671 | 2560 | 1719472.636 | 5.208 |
| Plane | Proportional | 256 | 6 | 1287.332 | 2560 | 1680741.209 | 2.621 |
| Plane | Proportional | 256 | 14 | 1272.968 | 2560 | 1661986.846 | 1.327 |
| Plane | Proportional | 256 | 30 | 1291.163 | 2560 | 1652688.496 | 0.678 |
| Plane | Proportional | 256 | 62 | 1287.644 | 2560 | 1648183.681 | 0.350 |
| Plane | Proportional | 256 | 126 | 1285.886 | 2560 | 1645933.596 | 0.177 |
| Plane | Proportional | 256 | 254 | 1285.000 | 2560 | 1644800.000 | 0.000 |
| Plane | Proportional squared | 510 | 0 | 1280.114 | 2560 | 262514277.899 | 0.506 |
| Plane | Proportional squared | 256 | 0 | 1280.465 | 2560 | 1802894.140 | 10.380 |
| Plane | Proportional squared | 256 | 2 | 1285.688 | 2560 | 3587582.693 | 5.208 |
| Plane | Proportional squared | 256 | 6 | 1279.833 | 2560 | 7044199.485 | 2.621 |
| Plane | Proportional squared | 256 | 14 | 1280.543 | 2560 | 13375014.776 | 1.327 |
| Plane | Proportional squared | 256 | 30 | 1280.746 | 2560 | 23606702.551 | 0.678 |
| Plane | Proportional squared | 256 | 62 | 1279.803 | 2560 | 36563458.045 | 0.350 |
| Plane | Proportional squared | 256 | 126 | 1279.801 | 2560 | 47538966.426 | 0.177 |
| Plane | Proportional squared | 256 | 254 | 1280.000 | 2560 | 52428799.937 | 0.000 |

**S2 Table. Parameterisation and power consumption details of networks simulated: a) branched networks, and b) random and radial burst networks**. The slope of the linear relationship between$\sigma_{P_{C}}$and $I^{2}$ shown in Fig. 1 was calculated for each plot using least-squares regression, and compared to the value of $\sigma_{R_{E}}$calculated using Eq. 5 and the consumer and resource potentials for each network, shown in the table here. The least-squares regression estimate of $\sigma_{R_{E}}$is shown in brackets below the original estimate using consumer and resource potentials, for the networks plotted.

**A.**

**B.**

| **Topology** | **Total network length** | **Node distribution** | **Resource potentials** | **No. of consumers** | **No. of resources** | **Mean consumer potential at maximum power** | **Mean resource potential at maximum power** | **Maximum power** | **$\sigma_{R_{E}}$** |
| --- | --- | --- | --- | --- | --- | --- | --- | --- | --- |
| Plane | 74877.080 | Random | Equal | 50 | 1 | 2501.289 | 5000.000 | 3251676.144 | 12.758  (12.760) |
| Plane | 415061.500 | Random | Equal | 100 | 1 | 4989.508 | 10000.000 | 11376078.579 | 3.249 |
| Plane | 1510034.000 | Random | Equal | 50 | 50 | 51.000 | 100.000 | 5864.995 | 4.974 |
| Plane | 3531541.000 | Random | Equal | 50 | 100 | 24.880 | 50.000 | 2114.770 | 2.898 |
| Plane | 1487595.000 | Random | Varied | 50 | 50 | 49.408 | 99.207 | 5681.931 | 4.602 |
| Plane | 3422254.000 | Random | Varied | 50 | 100 | 25.622 | 50.917 | 2177.879 | 2.941 |
| Plane | 11200.000 | Ring of consumers | Equal | 50 | 1 | 2491.200 | 5000.000 | 1395072.000 | 0.000  (0.000) |
| Plane | 31600.000 | Ring of consumers | Equal | 100 | 1 | 5007.200 | 10000.000 | 7911376.000 | 0.000 |
| Plane | 25228.310 | Ring of consumers | Equal | 50 | 10 | 2477.169 | 5000.000 | 619292.343 | 3.845  (3.845) |
| Plane | 25228.310 | Ring of consumers | Varied | 50 | 10 | 2508.767 | 5031.598 | 627191.844 | 70.955 |
| Plane | 25228.310 | Ring of resources | Equal | 10 | 50 | 99.093 | 200.000 | 990.925 | 0.000 |
| Plane | 25228.310 | Ring of resources | Varied | 10 | 50 | 98.335 | 199.261 | 983.353 | 5.802 |
| Plane | 4099.363 | Uniform random  (low connectivity) | Equal | 81 | 1 | 500.452 | 1000.000 | 1741572.000 | 0.387  (0.387) |
| Plane | 7680.167 | Uniform random  (med. connectivity) | Equal | 81 | 1 | 499.762 | 1000.000 | 4054065.000 | 0.102  (0.102) |
| Plane | 277.362 | Uniform radial | Equal | 81 | 1 | 500.056 | 1000.000 | 5768642.000 | 1.224  (1.224) |
| Sphere | 119384.300 | Random | Equal | 50 | 1 | 2517.610 | 5000.000 | 742694.886 | 6.084 |
| Sphere | 970045.300 | Random | Equal | 100 | 1 | 50006.146 | 100000.000 | 454555862.837 | 5.187 |
| Sphere | 2345486.000 | Random | Equal | 50 | 50 | 500.440 | 1000.000 | 357814.260 | 5.913 |
| Sphere | 5728683.000 | Random | Equal | 50 | 100 | 250.839 | 500.000 | 112877.518 | 4.952 |
| Sphere | 2321974.000 | Random | Varied | 50 | 50 | 501.006 | 1003.963 | 355714.309 | 7.556 |
| Sphere | 5766936.000 | Random | Varied | 50 | 100 | 249.526 | 501.682 | 121019.957 | 5.854 |
| Sphere surface | 214750.300 | Random | Equal | 50 | 1 | 24989.678 | 50000.000 | 40608226.348 | 16.033 |
| Sphere surface | 1216917.000 | Random | Equal | 100 | 1 | 49978.937 | 100000.000 | 525278626.787 | 13.582 |
| Sphere surface | 4600103.000 | Random | Equal | 50 | 50 | 497.706 | 998.014 | 194105.429 | 9.849 |
| Sphere surface | 10934860.000 | Random | Equal | 50 | 100 | 250.887 | 500.000 | 63976.129 | 5.982 |
| Sphere surface | 4607672.000 | Random | Varied | 50 | 50 | 498.283 | 1000.000 | 176890.508 | 13.087 |
| Sphere surface | 11111457.000 | Random | Varied | 50 | 100 | 253.070 | 501.161 | 62002.112 | 6.912 |
| Sphere surface | 8796.595 | Ring of consumers | Equal | 50 | 1 | 25000.077 | 50000.000 | 177625544.692 | 0.000 |
| Sphere surface | 24818.580 | Ring of consumers | Equal | 100 | 1 | 49990.557 | 100000.000 | 1007309730.737 | 0.000 |
| Sphere surface | 22606.750 | Ring of consumers | Equal | 50 | 10 | 2513.257 | 5000.000 | 691145.733 | 12.537 |
| Sphere surface | 22606.750 | Ring of consumers | Varied | 50 | 10 | 2493.034 | 5024.990 | 698049.528 | 58.146 |
| Sphere surface | 22606.750 | Ring of resources | Equal | 10 | 50 | 997.010 | 2000.000 | 110668.165 | 0.000 |
| Sphere surface | 22606.750 | Ring of resources | Varied | 10 | 50 | 996.453 | 1999.405 | 110606.287 | 16.571 |
