# Supplementary figures and images for: Trajectories toward maximum power and inequality in resource distribution networks
Source: PLoS One. 2020 Mar 10;15(3):e0229956. doi: 10.1371/journal.pone.0229956 (PMC7064246; doi:10.1371/journal.pone.0229956)

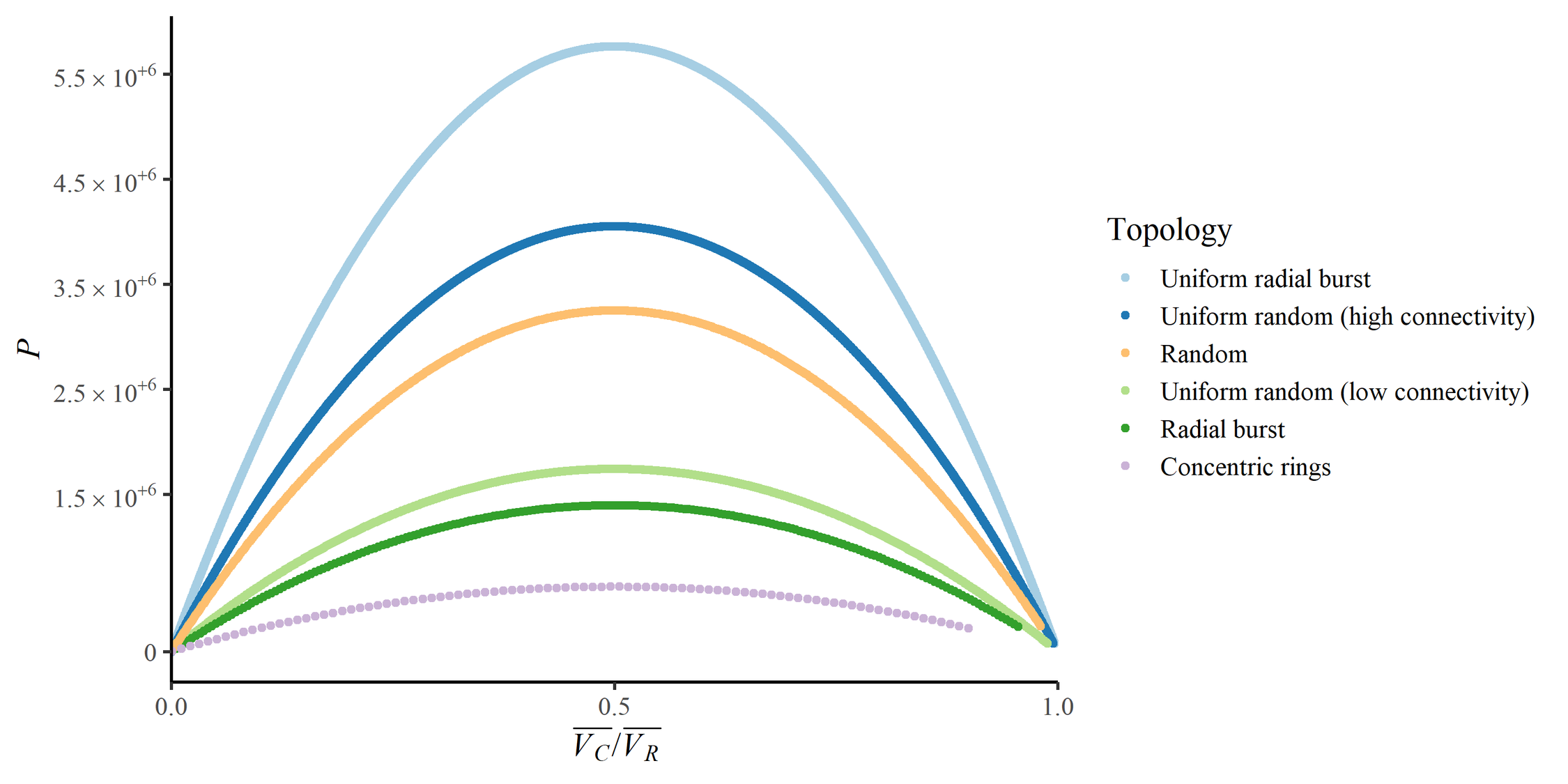

Supplement: S1 Fig — Each coloured point range represents a different network topology over which the simulations were run. The units are generalised units of power, rather than units only applicable to a specific type or types of resource distribution network. (TIF) [file pone.0229956.s003.tif]

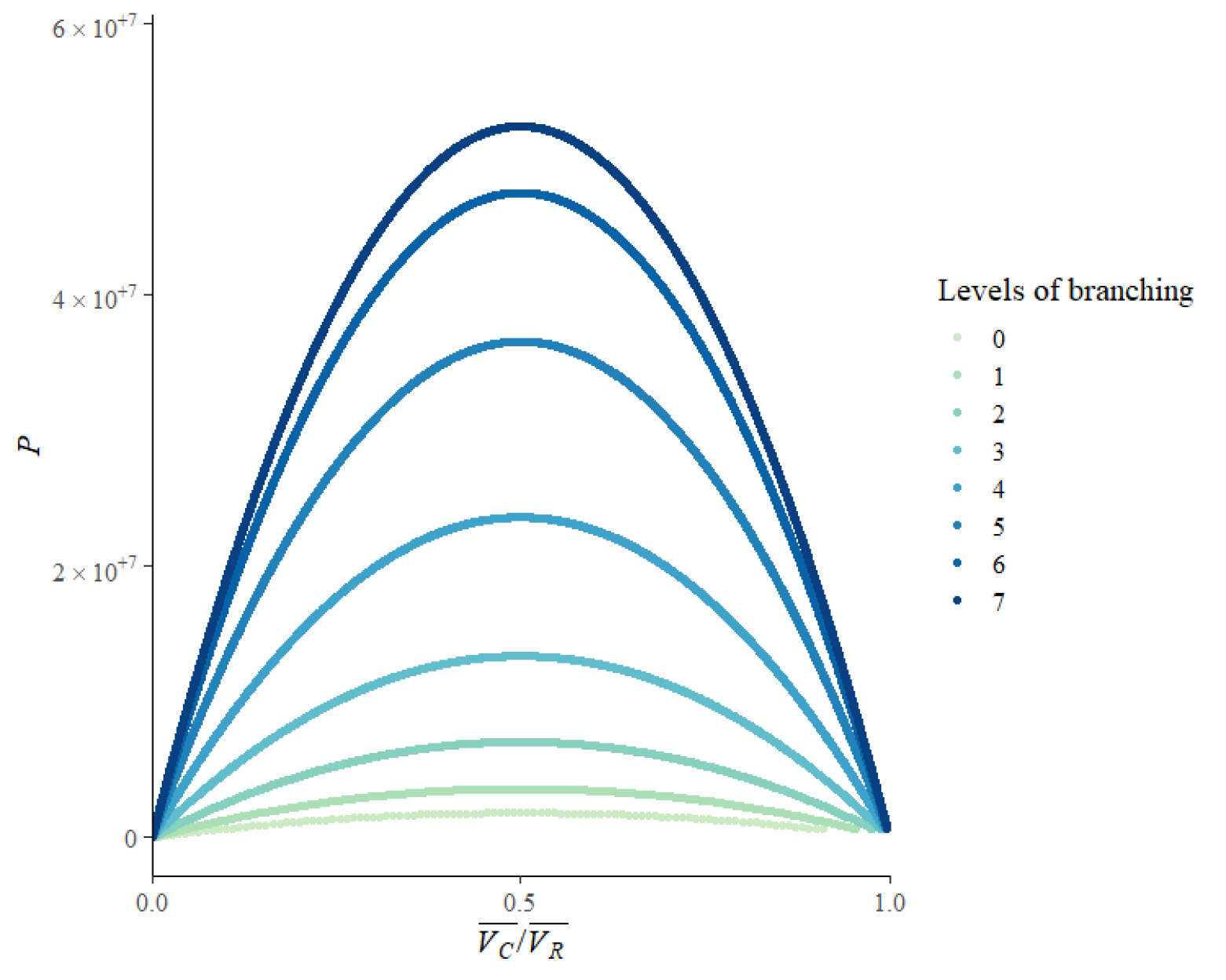

Supplement: S2 Fig — The units are generalised units of power, rather than units only applicable to a specific type or types of resource distribution network. (TIF) [file pone.0229956.s004.tif]

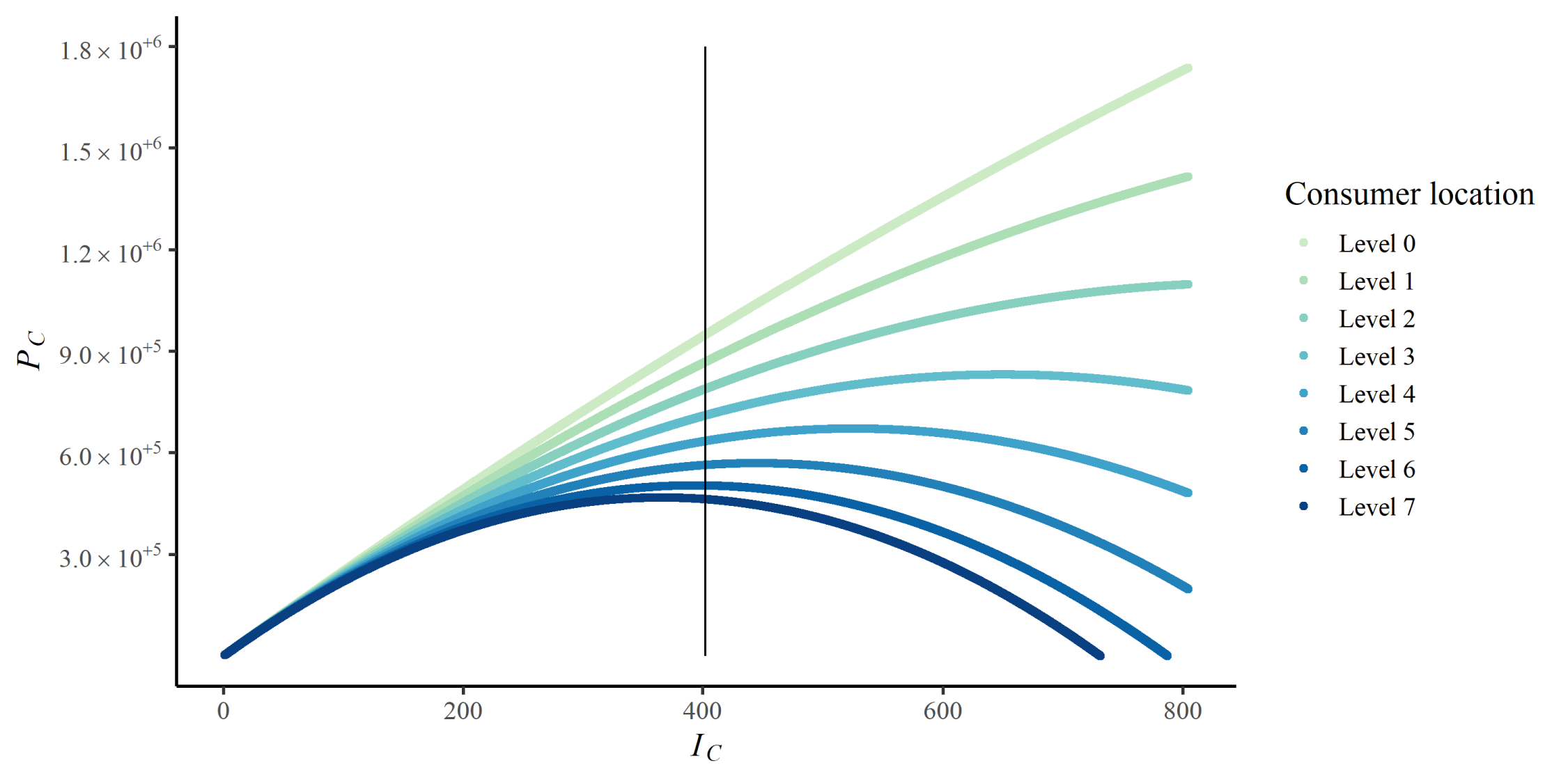

Supplement: S3 Fig — The units are generalised units of power and resource flow, rather than units only applicable to a specific type or types of resource distribution network. (TIF) [file pone.0229956.s005.tif]
